# Supplementary material for: MetaQTL: a package of new computational methods for the meta-analysis of QTL mapping experiments
Source: BMC Bioinformatics. 2007 Feb 8;8:49. doi: 10.1186/1471-2105-8-49 (PMC1808479; doi:10.1186/1471-2105-8-49)
Supplement: Additional File 7 — MetaQTL Package : jar file and tutorial. This Zip archive contains both the MetaQTL JAR file and the files of the tutorial. [file 1471-2105-8-49-S7.zip › org.inra.metaqtl/doc/org/metaqtl/class-use/MetaQtlAnalysis.html]

Uses of Class org.metaqtl.MetaQtlAnalysis


|  |  |  |
| --- | --- | --- |
| **Overview** \| **Package** \| **Class** \| **Use** \| **Tree** \| **Deprecated** \| **Index** \| **Help** | |  |
| PREV   NEXT | **FRAMES**    **NO FRAMES**     **All Classes** |  |


---


## **Uses of Class org.metaqtl.MetaQtlAnalysis**

| Packages that use MetaQtlAnalysis | |
| --- | --- |
| **org.metaqtl.adapter** |  |
| **org.metaqtl.algo** |  |
| **org.metaqtl.factory** |  |
| **org.metaqtl.main** |  |

Uses of MetaQtlAnalysis in org.metaqtl.adapter

| Methods in org.metaqtl.adapter that return MetaQtlAnalysis | |
| --- | --- |
| `static MetaQtlAnalysis` | `MetaQtlAnalysisAdapter.adapt(Chromosome[] chromosomes)` |

| Methods in org.metaqtl.adapter with parameters of type MetaQtlAnalysis | |
| --- | --- |
| `static QtlPartition` | `QtlPartitionAdapter.adapt(MetaQtlAnalysis analysis, java.lang.String chrom, java.lang.String trait, int k)`             Adapt the `MetaQtlAnalysis` `analysis` for a given value of the number of clusters `k` and a given trait name `code`. |

Uses of MetaQtlAnalysis in org.metaqtl.algo

| Fields in org.metaqtl.algo declared as MetaQtlAnalysis | |
| --- | --- |
| `protected  MetaQtlAnalysis` | `ClustAlgorithm.result`             The result of the clustering. |

| Methods in org.metaqtl.algo that return MetaQtlAnalysis | |
| --- | --- |
| `MetaQtlAnalysis` | `ClustAlgorithm.getResult()`             When the algorithm is finished use this method to get the result. |

Uses of MetaQtlAnalysis in org.metaqtl.factory

| Methods in org.metaqtl.factory that return MetaQtlAnalysis | |
| --- | --- |
| `static MetaQtlAnalysis` | `MetaQtlAnalysisFactory.read(java.io.InputStream stream)` |
| `static MetaQtlAnalysis` | `MetaQtlAnalysisFactory.read(java.io.Reader reader)` |

| Methods in org.metaqtl.factory with parameters of type MetaQtlAnalysis | |
| --- | --- |
| `static void` | `MetaQtlAnalysisFactory.write(MetaQtlAnalysis analysis, java.io.OutputStream stream)`             This methods writes out in the given `stream` the results of a QTL meta-analysis. |
| `static void` | `MetaQtlAnalysisModelFactory.write(MetaQtlAnalysis analysis, java.io.OutputStream stream)` |
| `static void` | `MetaQtlAnalysisSummaryFactory.write(MetaQtlAnalysis analysis, java.io.OutputStream stream)`             This method writes out the summary for a givene QTL meta-analysis result `analysis` on the given output stream `stream`. |
| `static void` | `MetaQtlAnalysisModelFactory.write(MetaQtlAnalysis analysis, java.io.Writer writer)` |

Uses of MetaQtlAnalysis in org.metaqtl.main

| Methods in org.metaqtl.main that return MetaQtlAnalysis | |
| --- | --- |
| `protected static MetaQtlAnalysis` | `MetaMain.getResult(java.lang.String resFile)` |

| Methods in org.metaqtl.main with parameters of type MetaQtlAnalysis | |
| --- | --- |
| `static ChromGraph[]` | `MetaMain.getChromGraph(Chromosome[] chroms, boolean withQtl, int modeQtl, IBioOntology ontology, MetaQtlAnalysis treeAnalysis)` |
| `static QtlPartition` | `MetaMain.getQtlPartition(Chromosome chrom, MetaQtlAnalysis analysis, java.lang.String trait, int k, char tclust)` |

---


|  |  |  |
| --- | --- | --- |
| **Overview** \| **Package** \| **Class** \| **Use** \| **Tree** \| **Deprecated** \| **Index** \| **Help** | |  |
| PREV   NEXT | **FRAMES**    **NO FRAMES**     **All Classes** |  |


---
